# Antechokinetics, the kinetics of antimicrobial resistance molecules

**DOI:** 10.3389/fphar.2026.1792355

**Published:** 2026-04-15

**Authors:** Fernando Baquero, Rafael Cantón, João Alves Gama, Jerónimo Rodríguez-Beltrán

**Affiliations:** 1 Departamento de Microbiología, Instituto Ramón y Cajal de Investigaciones Sanitarias (IRYCIS), Madrid, Spain; 2 Centro de Investigación Médica en Red - Epidemiología y Salud Pública (CIBERESP), Madrid, Spain; 3 Centro de Investigación Médica en Red – Enfermedades Infecciosas (CIBERINFEC), Madrid, Spain

**Keywords:** antechokinetics, antechology, antibiotic-resistance, antibiotic-resistance molecules, bacterial subcompartments

## Abstract

Antechokinetics is a related term to pharmacokinetics in which the focus shifts from drug kinetics to molecular kinetics of antibiotic-resistance. The term “antechó” is derived from the Greek word for “resistance.” The body is a multi-compartmental entity, and the bacterial cell is also composed of compartments. These compartments change much more rapidly than in a multicellular eukaryotic organism. There is currently a lack of knowledge regarding the antechokinetics of the molecules involved in antibiotic detoxification, but this is a field that requires further development. In this Review, we provide a summary of the current state of knowledge on the presence of antibiotic-resistance molecules in bacterial cells, focusing on their ribosomal production and eventual acquisition *via* exosomes or permeation. Next, we will examine their bacterial intracellular distribution, bioavailability, metabolism, and excretion. Future studies that consider the combined effects of antibiotic cellular pharmacokinetics and antechokinetics on a cellular level could be a fruitful area of research for the development of novel strategies to combat antibiotic-resistant infections.

## Introduction

Pharmacokinetics is a crucial part of pharmacology, focusing on how a specific drug molecule moves within the body. This includes the stages of absorption, distribution, bioavailability, metabolism, and excretion. In fact, pharmacokinetics is a particular case of what is studied in a general context by chemical kinetics theory. This theory specifically considers the motion of molecules or their trajectories in different compartments containing different types of matter with different structural consistencies. The concept of compartmentalization is paramount in the realm of chemical kinetics to living organisms. The human body is a skin-enclosed compartment made up of open (the respiratory, intestinal, and genitourinary tracts) and internal compartments (organs, connective tissue space, vascular and lymphatic systems, musculoskeletal structures) compartments, and the corresponding cellular microcompartments. Drug molecules in pharmacokinetics circulate among these compartments. The bacterial body is also composed of sub-compartments. However, intracellular bacterial pharmacokinetics is less well known than somatic pharmacokinetics in the host. In this review, we focus on a relatively understudied aspect of molecular transport between bacterial cellular subcompartments: the cellular transport of molecules that detoxify antimicrobial agents, thereby contributing to antimicrobial resistance. This emerging field of research, excluding the study of antimicrobial target mutations, was referred to as “antechokinetics” (AK), a term proposed by analogy with “pharmacokinetics” (PK). The term “antechokinetics” thus refers to the intracellular kinetics of antibiotic-resistance molecules within bacterial cells. Antecho” (αντέχω) is the Greek term for “resistance.” (Baquero et al., 2025). Antimicrobial resistance genes encode enzymes that produce various resistance molecules. First, primary effectors cause chemical breakdown (such as beta-lactamases) or alter the target molecule’s structure, thereby preventing toxicity (such as aminoglycoside-modifying enzymes). Second, secondary effectors modify antimicrobials at target sites (such as RNA methylases that protect ribosomes from macrolide inhibition) or induce the expression of primary effectors (like transcriptional activators of chromosomal beta-lactamases). The purpose of this study is to examine the applicability of well-established pharmacokinetic concepts and parameters used for drugs in animal and human models to molecules involved in antibiotic-resistance within bacteria. Although research in this area is limited, it may open unexpected opportunities for the development of new or modified antimicrobial agents. In antimicrobial therapy, antechokinetics cannot be separated from the intrabacterial pharmacokinetics of antibiotics (drugs). Overall, antechology (including antechokinetics and antechodynamics) represents a new epistemological framework within antimicrobial pharmacology.

## AK: incorporation of antibiotic-resistance molecules in the bacterial cell

Antibiotic-resistance molecules are present in bacterial cells as a result of local production, fusion with exosomes, acquisition through nanotubes, or permeation. In the host, this could correspond, by analogy, to the absorption phase in PK. The main difference is that antimicrobial resistance molecules are rarely acquired by bacterial cells from external sources. These elements are mainly produced as a consequence of the expression of intrinsic, chromosomal genes or of genes that reach the cellular cytoplasm *via* mobile genetic elements.

If these AK processes are recalled by analogy to the absorption phase in PK, in most cases, antimicrobial resistance molecules are not acquired by cells from the environment. These elements are produced as a consequence of the expression of intrinsic, chromosomal genes or of genes that reach the cellular cytoplasm *via* mobile genetic elements. Antimicrobial resistance genes are often intrinsic, forming the intrinsic resistome, which is frequently taxon-specific and includes environmental species. It is probable that these genes are, or have evolved from, gene ancestors involved in primary cellular functions unrelated to antimicrobial resistance. The detoxifying effect of the molecules encoded by these genes is a secondary consequence of their physiological effects on cellular processes (Baquero et al., 2021). To a certain extent, we can compare the acquisition and expression levels of these genes in the bacterial cell with the different drug concentrations resulting from various administration routes, as well as antimicrobial dosages in conventional PK.

The evolution of antibiotic-resistance mechanisms is also the result of bacteria living in complex environmental communities. These bacteria have evolved to defend themselves against the natural production of antibiotics by themselves or other bacteria in the community. This explains why the expression of intrinsic resistance genes and thus the production of antibiotic-resistance molecules is often regulated by global stress regulators, such as AmpR, a LysR transcriptional regulator of the chromosomal AmpC beta-lactamase. Small regulatory RNAs (sRNAs) have been shown to enhance the production of antibiotic-resistance molecules by regulating gene expression. They do so by controlling the translation or stability of target mRNAs. These target mRNAs are involved in various processes, including efflux pumps, cell envelope modification, nutrient uptake, and persister cell formation. Additionally, the repression of transmembrane pumps and drug uptake can be affected by alterations to the envelope, leading to a reduction in porin channels. Furthermore, sRNAs have been shown to enhance ribosomal synthesis of antimicrobial resistance molecules by either facilitating exposure of the ribosomal mRNA-binding site or by stabilizing mRNAs, thereby preventing their RNase degradation (Yang et al., 2025). The field of research on the role of ribosomal protein network dynamics in the exposure of the binding sites (Timsit et al., 2025) shows great promise for understanding the production levels of resistance molecules.

The concentration of antibiotic-resistance molecules in a cell is influenced by the number of antibiotic-resistance encoding genes present, as well as the number of ribosomes available. In the first case, it is the known “gene-dosing effect.” Such an effect is the result of over-synthesis of proteins with weak detoxifying activity and/or from stoichiometric relations between mechanisms of resistance and antibiotic substrates. Gene-dosing effect, starting with reversible gene tandem amplification, is frequently an effective way of adaptation to antibiotic exposure (Martinez et al., 1989a; Sandegren and Andersson, 2009). Increased gene copy dosage is derived from two sources: tandem amplification and an increase in the copy number of genetic elements carrying resistance genes. Small plasmids often have a high number of copies, a phenomenon that was previously explained by their stochastic effects on antibiotic-resistance (Martinez et al., 1989b; Hernandez-Beltran et al., 2024). Transposition of resistance genes to small cryptic plasmids has also been shown to increase the gene copy-number (Nicoloff et al., 2024). The number of plasmids per cell varies significantly among bacterial species, with a range of approximately three orders of magnitude, depending on the plasmid size and the host genome size. Gram-negative bacteria (Pseudomonadota) typically have more small copy number plasmids than Gram-positive (Bacillota) (Ramiro-Martínez et al., 2025). According to Dubey and Ben-Yehuda (2011) and Tran and Boedicker (2019), nanotubes and diffusible extracellular vesicles can serve as intercellular carriers of small plasmids. These vesicles originate from the blebbing of a weakened peptidoglycan layer in the bacterial envelope, and the loading of plasmids into vesicles correlates with the plasmid copy number (Biller et al., 2025). These extracellular vesicles can also directly convey the acquisition of antibiotic-resistant proteins, mimicking the external administration of antimicrobial drugs. This process may involve adhesion-fusion with the bacterial envelope of extracellular vesicles originating from antibiotics, predators, or phage-mediated cellular explosion, predominantly in Gram-negative cells. Simpler outer membrane vesicles might contain resistance molecules, such as periplasmic beta-lactamases, frequently found in biofilm-living bacteria (Jiang et al., 2024; Martínez et al., 2021; Ciofu, 2003).

The synthesis (dosage) of plasmid- and chromosomal-biomolecules involved in antimicrobial detoxification is proportional to the number of functional ribosomes in the cell. For instance, *Escherichia coli* can be killed by bacteriostatic antibiotics such as tetracycline, chloramphenicol, or azithromycin by reducing the number of *rrn* operons (Levin et al., 2017). It is important to note that a high ribosomal processivity, defined as the probability that a ribosome that has initiated translation on an open reading frame will complete elongation, may assure effective translation of antibiotic-resistance genes.

Direct acquisition of antibiotic-resistance molecules from external sources is rare or nonexistent, with the exception of microvesicles and nanotubes previously mentioned (Pande, S., Shitut, S., Freund, L. *et al.* Metabolic cross-feeding *via* intercellular nanotubes among bacteria. *Nat Commun* 6, 6238 (2015). In the case of antimicrobial agents that act as intracellular antimetabolites, increased uptake of the target molecule from the external environment can serve as a resistance mechanism. Examples of this phenomenon include cases of resistance to sulfonamides or trimethoprim due to exposure to folates produced in excess by neighboring bacteria, such as lactic acid bacteria (Wegkamp, 2008). Similarly, exposure to thymine or thymidine originating from DNA-RNA degradation of neighboring dead cells may reduce the activity of the antifolate inhibitor.

## AK: distribution

AK-Distribution refers to the process by which a resistance biomolecule is dispersed throughout the bacterial cellular compartments. This definition mirrors the PK-biodistribution in the blood and tissues of animals, including the human body (Onetto and Sharif, 2025). A limitation is that the cellular compartments in bacteria are frequently less well-defined. However, the bacterial cytoplasm is an enveloped hydrogel crowded with a multiplicity of organelles and molecules occupying distinct spaces, giving rise to a subcellular architecture (Baquero et al., 2023). It should be noted that the size and shape of these compartments can exhibit temporal organization. That is to say, their volume and density can change over time, particularly during growth phases, planktonic or biofilm-style, or when cells are submitted to changes in osmolarity or other stresses, including the smaller cells of “persisters” (Valencia-Burton et al., 2009; Trevors, 2012; van den Bogaart et al., 2007).

**FIGURE 1:**
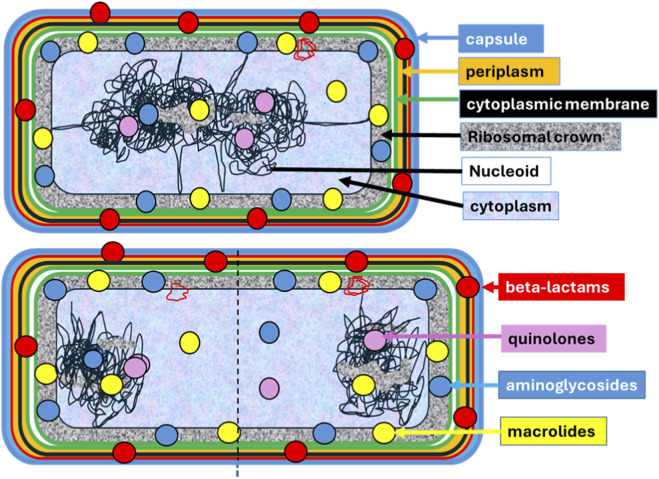
Schematic representation of the subcellular compartments in a Gram-negative bacteria. In the top cellular representation, the main compartments are listed in the legends on the right. From outside to inside, in blue, lipopolysaccharide layer; in orange, periplasmic space, limited by the external layer in red of external membrane, and including the cell wall in black; in green, the bilayered cytoplasmic membrane; in dotted grey, a densely ribosome populated space, the ribosomal crown; in light blue, the cytoplasm; diffuse cytoplasmic filaments are in rose; the black line forming a twisted mesh represent the nucleoid, where ribosomes (dotted grey) can be also present. Note that nucleoid filaments may approach the ribosome crown. In the diagram below, the color code for different antibiotic-resistance molecules is shown in the legends on the right; it also applies to the top cellular schema. Here, the cell is elongated to represent the upcoming division; the nucleoids have been segregated into the space of the future daughter cells, and nucleoid sectors may be closer to the ribosomal crown.

The cellular envelope in Gram-negative bacteria has a well-defined compartment, the periplasm. In Gram-positive bacteria, we can consider an equivalent space mainly containing the thick peptidoglycan and the associated teichoic acids. There is a cytoplasmic membrane space associated with a densely populated proteomic sacculus. Structural oligomeric complexes facilitate interaction between the cytoplasmic membrane and the 25% of the total cell proteome. The membrane space is closely associated with a “ribosomal crown”, a sub-cytoplasmic wide and dense layer of ribosomes that constitutes the protein-factory compartment. This compartment contains the majority of the cellular ribosomes, which number about 50,000, mostly polyribosomes. In a centripetal direction, there is a contiguous compartment that is less rich in ribosomes. The nucleoid space occupies 10%–20% of the bacterial cell volume and contains a free and bound ribosomal population. It has been estimated that the nucleoid contains 20% of the total cell ribosomes (Bakshi et al., 2012). However, in growing cells, the relatively free perinucleoid space is dynamically crossed by pulled-out, extended chromatin excrescences originating in the nucleoid and reaching the sub-membrane ribosomal crown (Robinow and Kellenberger, 1994). This is likely necessary due to the brief duration of both gene transcription and the mRNA transcript half-life, which are approximately 20 s and 5 min, respectively, in the case of *E. coli*. This necessitates swift interaction between the nucleoid and the ribosome crown to facilitate expeditious translation (a process known as coupled transcription-translation, or transertion). Alternatively, in regions of the highest curvature, a solid-sphere-like nucleoid can be in close proximity to the membrane-associated ribosomal crown (Joyeux, 2019). A schematic representation of the topology of this compartmental structure is shown in Figure 1.

## AK: bioavailability

Bioavailability refers to the proportion of produced antimicrobial resistance molecules that reach and detoxify the antimicrobial molecules, or that provide protection for these targets. This definition aligns with PK-bioavailability, which considers the amount of drug administered (Price and Patel, 2025). As outlined in the bacterial compartment model, the majority of chromosomally encoded antibiotic-resistance proteins should be produced and reach high concentrations in the ribosomal crown near the cytoplasmic membrane. Please note that this does not exclude the translation of mRNAs in ribosomes associated with the nucleoid. A key question is whether the compartments with a higher concentration of the antimicrobial resistance biomolecules are also rich in antimicrobial drugs after therapeutic dosage. Local stoichiometry conducive to the synthesis of resistance biomolecules should enhance resistance efficiency. The most well-known example is the presence of beta-lactamases in Gram-negative bacteria. These enzymes are translated as a pre-beta lactamase in the cytoplasm, carrying an N-terminal leader signal recognized by the Sec (secretion) system. This allows the molecule to be translocated across the cytoplasmic membrane and concentrated in the periplasmic compartment (Kaderabkova et al., 2022; Driessen and Nouwen, 2008).

In some cases, the beta-lactamase might remain associated with the membrane compartment (Plückthun and Pfizinger, 1988). The extra-periplasmic location of beta-lactamases is also a possibility. These enzymes may be retained in the outer membrane, the capsular material, or excreted outside the cell, typically in Gram-positives. As is well known in the industry, beta-lactam antibiotics can access the periplasm in Gram-negatives from the external environment, mainly *via* outer membrane proteins that serve as uptake channels (porins). Beta-lactamases, which are present in this compartment, can efficiently hydrolyze the drugs, thereby protecting the cell wall in this space.

Research indicates that even if the classic feeling was that aminoglycoside resistance molecules, including both aminoglycoside modifying enzymes and methyltransferases, as well as the S-adenosyl-L-methionine acting as a methyl donor, were randomly distributed in the cell cytoplasm, they should be mostly located in the ribosomal crown compartment near the cytoplasmic membrane. During the process of uptake, aminoglycosides bind to negatively charged membrane phospholipids or are readily associated with nucleic acids, particularly mRNA and rRNA (Arya et al., 2005; Zarubica et al., 2011). Therefore, there is an expected neighborhood between antibiotic-resistance molecules and drugs, but antibiotic-modifying enzymes are probably inactive against aminoglycosides trapped in the membrane, although this complex can eventually release the antibiotic during bacterial growth phases. The quinolone-resistance protein Qnr has been found to be natively associated with DNA repair (Gil-Marqués et al., 2021), suggesting that its transcription is likely to be exerted in the DNA-associated ribosomes. Ribosome-inhibiting antibiotics, such as tetracyclines, chloramphenicol, macrolides, lincosamides, and streptogramins, are likely to be detoxified by proteins translated in neighboring ribosomes. In this region, resistance molecules and antibiotic targets also co-localize.

Plasmids contain transposons and integrons with a wide variety of antibiotic-resistance genes, so the distribution of plasmids in the bacterial cytoplasm and cytoplasmic membrane, and their interactions with ribosomes, should be considered. It is well established that different plasmids are located in precise sites within the bacterial cell. However, it is also known that these plasmids move from one location to another during the cell replication cycle. Multicopy plasmids often coalesce in clusters during the process of intracellular migration. This process is facilitated by the subcellular localization of partitioning proteins, which has provided significant insights into the mechanisms of DNA movement within the cell. This is primarily achieved by tagging individual DNA molecules with green fluorescent protein (Pogliano, 2002). The required interaction between plasmids and ribosomes appears to be a manageable challenge, given the relative numbers of both entities. For instance, the ratio of plasmids (2–200) to ribosomes (50,000–70,000) in *E. coli* cells is 2:1, which is favorable for the former.

Despite the anticipated frequent co-localization of antibiotics and antibiotic-resistance proteins within bacterial compartments, a question remains unanswered. This concerns the possibility of unknown mechanisms whereby specific mRNAs encoding antibiotic-resistance molecules are directed to ribosomes in specific compartments, while others are stochastically mobilized (Trevors, 2012). This can also be applied hypothetically to antibiotic-resistance molecules. Are they targeted to the locations where antibiotics are having an adverse effect? For instance, subcomponents of the bacterial structure might act as signals influencing the synthesis of resistance proteins (Baquero et al., 2026). Recent findings contradict the long-standing view that proteins, including those with antibiotic-resistance, diffuse randomly in the cytoplasmic viscous hydrogel (van den Bogaart et al., 2007). This conclusion is based on a study that examined the fluorescence recovery of labeled proteins after photobleaching. There are three main types of diffusion: Brownian, random, and confined (Kapanidis et al., 2018). The latter type is characterized by bursts of diffusion along a specific path. In addition, directed motion can be traced through straight or angled lines, suggesting a trajectory. Diffusion may depend on protein net charge density, surface hydrophobicity, and electric dipole moment (Mu et al., 2017). Protein directional movements may be influenced by cytoplasmic filament systems (Eun et al., 2015).

Integrating all these observations and predictions, a framework for the intracellular bioavailability, in our case, applicable to antibiotic-resistance molecules within bacterial cells, has been proposed to model intracellular reaction kinetics. It takes into account the physical nature of the intracellular compartment in which it occurs, the typical chemical concentrations involved in the reaction, and the average intermolecular distances, deduced from the diffusion coefficients, reaction constants, and reactant concentrations. These parameters approximate the deterministic or stochastic nature of molecular interactions (Grima and Schnell, 2008).

A significant gap in the development of antechokinetics is the lack of observational and experimental data on the concentrations of antibiotic-resistance molecules and the numbers of antibiotic molecules to be detoxified across different compartments. The volume of distribution, maximal concentrations, and half-times of both reactants are challenging to estimate with the available technology. These numbers can vary significantly among bacterial types and growth phases, planktonic or biofilm lifestyle, cell shape under stress, and measurement method (Dumont et al., 2019). The objective of future research is to ascertain the effective detoxifying concentration for the various resistance biomolecules under different therapeutic conditions.

## AK: metabolism

According to the established definition, AK-Metabolism signifies the process by which a protein involved in antimicrobial resistance is converted within the bacterial cell into subsequent compounds, often resulting in the loss of its activity (Grogan and Preuss, 2022). There is a limited understanding of the potential intracellular degradation of antibiotic-resistance proteins. The natural bacterial protein turnover (proteostasis) could be considered a “clearance” mechanism for resistance proteins during stationary phase. Intracellular proteolysis, carried out by energy-dependent proteases, could be expected to degrade these proteins. Evidence remains limited. Bacterial proteases, including the main ATP-dependent protease ClpXP, are not effective at degrading pre-beta-lactamases, beta-lactamases, or other resistance proteins. (Zou et al., 2021). The inactivation of ClpXP protease appears to increase MRSA beta-lactam resistance, but this may be related to the chaperone function of this molecule, not to the protease (Bæk et al., 2014). However, proteolysis-targeting chimeras, BacPROTACs (Morreale et al., 2022) have been shown to degrade resistance factors in Gram-positive pathogens. Additionally, the ClpXP mechanism has been observed to degrade the β-lactamase CTX-M-14 (Nie et al., 2025). However, proteolytic processes are significant in the evolution of beta-lactamases. In the case of metallo-beta-lactamases (as NDM-1), the non-metallated precursor is degraded by the periplasmic protease Prc. The binding of Zn(II) renders the protein refractory to protease degradation (González et al., 2023).

There is currently no clear evidence that the bacterial phosphoproteome plays a direct role in the inactivation of antibiotic-resistance molecules (Bäsell et al., 2014). However, in the case of *Acinetobacter baumannii*, the process of phosphorylation of the AmpC β-lactamase has been shown to reduce its hydrolyzing activity against imipenem (Lai et al., 2016). Although there is a lack of reports on the phosphorylation of Gram-positive β-lactamases associated with detoxification, there is a recent example of the beta-lactamase BlaZ from *Staphylococcus aureus* that should be phosphorylated as a prerequisite to its sequestration on the cytoplasmic membrane, where its hydrolytic activity against β-lactams is retained (Kim et al., 2025). Phosphorylation cascades may indirectly influence the level of resistance of certain antibiotics *via* two-component systems. One example of this is colistin resistance, which modifies the target lipopolysaccharide. However, this protein is not altered in the cell envelope (Wang et al., 2024). This lack of direct effect on resistance biomolecules is also the case in the bacterial acetylome. Research indicates that the degree of protein acetylation may modify the level of antibiotic action. However, this effect appears to be more related to changes in cellular metabolic activity than to modifying the resistance molecule (Fang et al., 2022). However, *Streptomyces verticillus* produces bleomycin, which offers it self-protection through bleomycin N-acetylation (Chen et al., 2022). The bleomycin-sequestration protein, encoded by the *ble* gene, has been inserted into the transposon Tn*5* (Kumagai et al., 1999).

Resistance biomolecules are typically present in bacterial organisms that produce antimicrobials and serve as self-protection mechanisms (Cundliffe, 2007; Tenconi and Rigali, 2018). To ensure immediate co-localization of the antibiotic and the resistance mechanism, resistance genes are generally clustered and co-controlled with the genes involved in antibiotic biosynthesis. For instance, this is the case for *Streptomyces clavuligerus*, which produces the β-lactam antibiotic cephamycin C (Paradkar, 2013).

## AK: excretion

In PK, excretion refers to the clearance rate of a drug from the body, frequently in a complete form. Nothing similar occurs in AK, as bacteria are the main source of the resistance mechanisms. According to the findings of early studies, 40% of *S. aureus* beta-lactamase BlaZ was released in the growth medium (Coles and Gross, 1967). As previously mentioned, the primary cause is BlaZ sequestration in the bacterial cytoplasmic membrane and surface. The Bla-Z and also the NDM-carbapenemase lipidation ensure anchoring to membrane phospholipids (Nielsen et al., 1981; Smisek et al., 2025). The antibiotic-resistance molecules that are actively excreted from bacteria, such as penicillinases in *S. aureus*, do not lose their function, unlike drugs outside the body. Lysed bacterial cells also release antibiotic-resistance molecules into the close peri-bacterial spaces. This may suggest the possibility of studying extracellular antechokinetics, how bacterial-originated antibiotic-resistance biomolecules diffuse in environmental compartments, eventually protecting neighboring bacteria from antibiotics, but this topic is not unrelated to the purpose of the present review, focused on intracellular antechokinetics.

## Conclusions and perspectives

The therapeutic action of antimicrobial agents on microorganisms triggers a response in the microbial world, frequently mediated by antibiotic-resistance biomolecules. The effectiveness of antibiotics in treating infectious diseases depends on their pharmacodynamics and pharmacokinetics. These factors are taken into consideration in both therapeutic guidelines and the design of new antibiotics. Conversely, the effectiveness of antimicrobial resistance mechanisms mediated by biomolecules that detoxify antibiotics hinges on the likelihood of intermolecular reactions between the antibiotic and the resistance molecule. The antibiotic’s mechanism of action involves movement within the bacterial cell, passing through different cellular subcompartments. The details, particularly the quantitative aspects of antibiotic cellular pharmacokinetics in the changing biology of bacteria, are only partially known. The kinetics of the biomolecules involved in antibiotic-resistance within bacterial cells are also poorly understood. It is critical to understand the interactions between pharmacokinetics and antechokinetics within bacterial cell subcompartments. To reach a quantitative antechokinetics with similar parameters to those used in pharmacokinetics (Cmax, Vd, t1/2) in these bacterial subcompartments is a hard task. The size and composition of such subcompartments, vary in response to microecological changes in host tissues, including those associated with pathogenicity, such as pH, temperature, osmolarity, cytokines, superoxides, or available nutrients (Baquero et al., 2023). It is thus expected that a range of antechokinetic variation occurs across different situations and antibiotic classes, as well as among resistance molecules. However, we are obliged to invest in research to quantify antibiotic-resistance biomolecules. Advanced cellular imaging technology may be useful to reach such a goal, including live-bacterial cell fluorescence microscopy (Gitai, 2009; Schneider and Basler, 2016), probably in combination with cryogenic electron tomography (Navarro et al., 2022). Mathematical modeling is a powerful promising strategy to combine these kind of parameters (de Jong et al., 2017). Modeling antechokinetics should also be possible by applying membrane-computing technology, in which the different subcellular compartments can be represented, as well as the variable rules governing transmission of molecules across these spaces (Baquero et al., 2018; Campos et al., 2019). Finally. Artificial Intelligence and Machine Learning procedures may contribute to refine the quantification of antechokinetics parameters (Gharat et al., 2024).

In addition, other unexplored parallels in the study of drugs and antibiotic-resistance determinants warrant consideration, such as drug toxicity in the host and the fitness costs imposed on bacterial cells by the expression of mechanisms of resistance in host environments (Baquero et al., 2024). As pharmacokinetics has contributed to the discovery of new, more effective antimicrobials, antechokinetics may also elucidate key aspects of the intracellular transmission of antibiotic-resistance molecules. That will inform the design of innovative antimicrobial agents, antibiotic-resistance inhibitors, adjuvant molecules, or intracellular microecological modifiers that can disrupt the interactions between antibiotics and resistance mechanisms to control infectious diseases caused by antibiotic-resistant microorganisms.
